# An Evaluation of Graphene Oxides as Possible Foam Stabilizing Agents for CO_2_ Based Enhanced Oil Recovery

**DOI:** 10.3390/nano8080603

**Published:** 2018-08-08

**Authors:** Albert Barrabino, Torleif Holt, Erik Lindeberg

**Affiliations:** 1 Petroleum Department, SINTEF Industry, NO-7465 Trondheim, Norway; torleif.holt@sintef.no; 2 CO_2_ Technology, NO-7030 Trondheim, Norway; erik.lindeberg@sintef.no

**Keywords:** enhanced oil recovery, graphene oxide, CO_2_ foam, aquifer storage, mobility control

## Abstract

Graphene oxide, nanographene oxide and partially reduced graphene oxide have been studied as possible foam stabilizing agents for CO_2_ based enhanced oil recovery. Graphene oxide was able to stabilize CO_2_/synthetic sea water foams, while nanographene oxide and partially reduced graphene oxide were not able to stabilize foams. The inability of nanographene oxide for stabilizing foams was explained by the increase of hydrophilicity due to size decrease, while for partially reduced graphene oxide, the high degree of reduction of the material was considered to be the reason. Graphene oxide brine dispersions showed immediate gel formation, which improved foam stability. Particle growth due to layer stacking was also observed. This mechanism was detrimental for foam stabilization. Gel formation and particle growth caused these particles to block pores and not being filterable. The work indicates that the particles studied are not suitable for CO_2_ enhanced oil recovery purposes.

## 1. Introduction

CO_2_-based methods for enhanced oil recovery (EOR) in water flooded reservoirs faces various technical challenges. The lower density of CO_2_ compared to water causes gravity segregation and the lower zones of the reservoir will not be swept by CO_2_. The low CO_2_ viscosity leads to viscous fingering and excessive flow in high permeability layers. The net effect can be an early CO_2_ breakthrough, reduced sweep efficiency and low oil recovery. Counteracting these effects can be achieved by decreasing the CO_2_ mobility, either by adding thickeners into CO_2_ or dispersing it into brine (CO_2_ foam) [1,2,3,4].

Direct thickeners CO_2_ require molecules that are CO_2_ soluble and have groups that interact giving the increased viscosity. Efforts to develop thickeners have been ongoing through the last decades. Up to now, the best results have been obtained with a fluoroacrylate-styrene copolymeric thickener that at typical reservoir conditions is able to increase the CO_2_ viscosity by the order of ten using low concentrations (<1 wt. %) [1]. However, due to costs and environmental concerns, these types of additives are unlikely to have a practical application.

The apparent viscosity of CO_2_ dispersed into foams may be very high, depending on the type of surfactant used. One potential problem is that many foams are sensitive to the presence of oil giving destabilisation through several mechanisms including spreading and entering phenomena [5,6,7]. During miscible CO_2_ flooding, oil sensitivity may be an advantage as foam formation is desired where the oil already has been displaced, diverting the CO_2_ into the oil containing parts of the reservoir. However, also minute amounts of oil remaining after miscible flooding may have detrimental effects on foam propagation [8].

CO_2_ foams can also be stabilized by nanoparticles. Nanoparticles adsorb strongly at interfaces which contribute to a higher stability. However, the mixing energy required to adsorb at interfaces is larger than traditional surfactant stabilized systems. This is an important disadvantage for oil recovery [9,10,11,12]. Typically, oil field flow velocities do not exceed a few feet/day which results in low mixing energy. It may, therefore, be difficult to utilise nanoparticle stabilized foams under normal process conditions.

The use of binary mixtures of surfactant and nanoparticles may improve foamability at low flooding rates. Singh et al. [13] showed that a mixture of anionic surfactant and fly ash could reduce CO_2_ mobility more than anionic surfactant alone. Cationic surfactant had the opposite effect, however. Manan et al. [14] demonstrated that mixtures of surfactant and different types of nanoparticles improved oil recovery during CO_2_ flooding compared to only surfactant, the improvement depended on the type of particles used.

Patel et al. [15] studied oil-in-brine emulsion stability of silica nanoparticles in presence of sodium dodecyl sulphate. The presence of surfactant allowed to increase suspension stability by diminishing particle flocculation. Even though they observed that nanoparticles were more effective for stabilizing oil emulsions, binary mixtures of particles and surfactant were detrimental for emulsion stability compared to only nanoparticle-stabilized emulsions. These observations are consistent with the competitive adsorption conclusion of Pichot et al. [16].

A binary system with surfactant and nanoparticles will be vulnerable to the separation of the constituents during transport in porous media and beneficial system properties may thus be lost. Ideally, the foam stabilising agent should be uniform to avoid loss of performance. To find foam stabilising agents with a uniform composition having the desired properties (sufficient mobility reduction, the desired level of oil sensitivity, low loss, moderate cost, etc.) remains a challenge.

Graphene Oxide (GO) is a relatively new material which is being widely studied due to its characteristics and its relevance to a wide range of fields, such as energy materials, biosensors, catalysis and biomedicine [17,18,19]. GO particles have shown to be surface active with a size-depending amphiphilicity and they have been reported to create very stable emulsions with organic solvents [20]. The higher hydrophilicity of smaller particles is attributed to a higher density of –COOH on its edges and epoxy groups on its surface [21]. Having all this under consideration, GO amphiphilicity can be tuned by variations on its size [21] or by partially reducing the particles [22].

Recently, Liu et al. [22] have reported for the first time that partially reduced graphene oxide (rGO) can efficiently stabilize CO_2_ in water. Foam stability was explained by the large surface area of GO, which diminished contact between CO_2_ and water. Moreover, Liu et al. [23] demonstrated that GO can be an effective demulsifier for heavy oil-in-water emulsions. This effect was attributed to the strong interactions between the GO nanosheets and the molecules of asphaltenes and resins.

GO is a candidate for application within the oil industry. However, studies for its applications in EOR has up to now not been published. In this work, we have studied the possible use of GO/rGO particles as foam stabilizing agents for CO_2_ EOR.

In this article dispersions of aqueous solutions and CO_2_ are being referred to as foams whereas dispersions of aqueous solutions and organic solvents are named emulsions.

## 2. Experimental

### 2.1. Materials

GO and rGO materials were supplied by Graphenea S.A., San Sebastián, Spain. GO was delivered in a water suspension with a total concentration of 4 mg/mL. The particle size was polydisperse, ranging from 4 to 30 µm. GO suspensions with a smaller particle size (nGO) were also provided by Graphenea S.A. The nGO was prepared by GO long-time ultrasonication treatment done by the supplier. The nGO particle size was determined using a Zetasizer Nano ZS (Malvern Instruments Ltd., Malvern, UK) after being ultrasonicated and filtrated through a 0.45 µm syringe filter. rGO was obtained in solid powder form. The particle size of rGO ranged from 260 to 295 nm as specified by the provider. The elemental composition of both materials was also obtained from the provider and is described in Table 1. The synthetic sea water (SSW) composition used for all experiments is shown in Table 2.

The organic solvents used for the initial stability tests were: toluene, obtained from MERK (99.5%), n-hexane, supplied by VWR (99%) and n-decane from MERK (99%).

### 2.2. Methods

#### 2.2.1. Bottle Test

The bottle tests were carried out with a volume fraction of 50% of each phase (6 mL of total volume), SSW and organic solvents. The particle concentrations used were 1 mg/mL and 0.5 mg/mL GO in the SSW. The mixture was shaken by hand vigorously for 10 s and placed in a graduated glass test tube where the volumes of each phase were read.

#### 2.2.2. Phase Equilibria Studies

**Figure 1:**
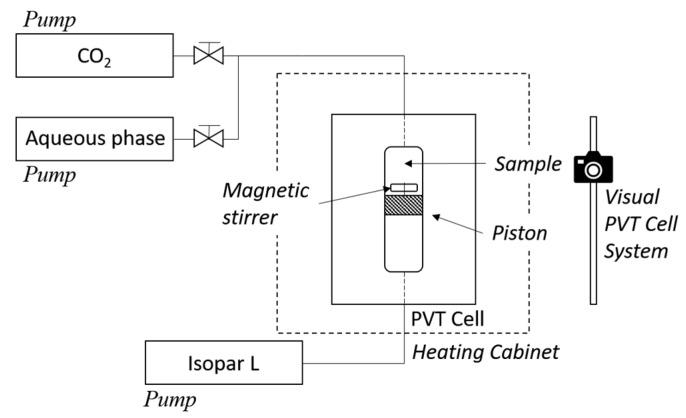
Schematic representation of the pVT cell used in the experiments.

An internally stirred windowed high-pressure, high-temperature pVT-cell allowing visual observation along the whole volume of the cell was used for the measurements (Figure 1). The cell was placed inside a temperature-controlled heating cabinet. Equal volumes of dense CO_2_ and aqueous solutions were injected into the cell by using high-pressure pumps and the pressure was adjusted to the initial test-pressure. The total test volume was approximately 60 mL. The pressure varied typically less than 2% during the tests.

The foams were created using a magnetic stirrer located inside the cell. The system was stirred for one minute. During stirring, the cell was tilted 180 degrees vertically one time and returned to its original position for favouring phase contact. After the stirring stopped the phase heights (CO_2_, foam, SSW) were determined over time using a cathetometer.

## 3. Results and Discussion

### 3.1. Emulsion Stability

**Figure 2:**
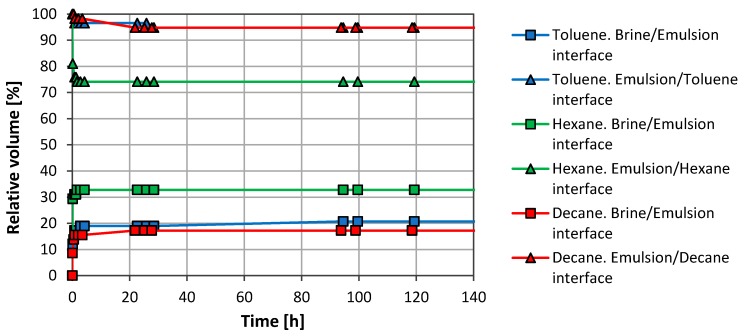
Emulsion stability for SSW/solvent systems. The GO concentration used was 1.0 mg/mL in the aqueous phase and the oil fraction was 50 vol. %.

Initial tests with organic solvents were carried out just to observe the GO ability to stabilize organic solvent/water emulsions. Figure 2 shows both separate on profiles, oil phase/emulsion and emulsion/aqueous phase, for each system with a concentration of 1 mg/mL GO in the aqueous phase.

All systems showed good stability and, after the separation observed during the first minutes, the emulsions remained stable for weeks. Toluene and n-decane had a similar emulsified volume, while hexane showed a lower emulsion volume (~41 vol. %).

The samples were immersed in a water bath to study the temperature effect on emulsion stability at 30 °C, 50 °C and 80 °C. No differences were observed between room temperature, 30 °C and 50 °C. However, at 80 °C larger droplets of solvent were observed trapped inside the emulsion phase and there was a slight solvent phase volume increase.

**Figure 3:**
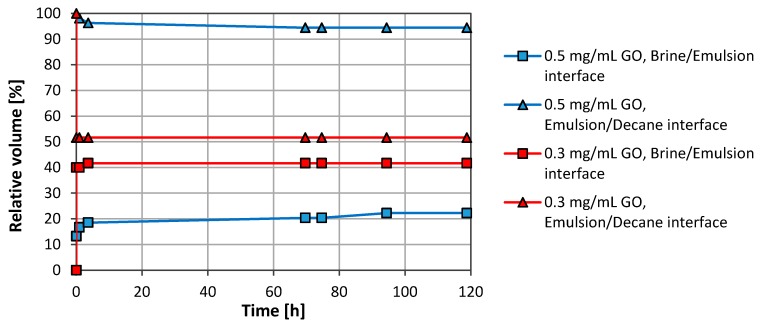
Effect of particle concentration on emulsion stability. N-decane was used as the oil phase, 50 vol. %.

The effect of the particle concentration was also studied using concentrations ranging from 0.1 to 1.0 mg/mL. Figure 3 shows results for 0.3 mg/mL and 0.5 mg/mL. The stability for 0.7 mg/mL and 1.0 mg/mL was as for 0.5 mg/mL. However, there was a drop in the stability when the concentration was reduced from 0.5 to 0.3 mg/mL. The results obtained with 0.1 mg/mL were almost similar to the results obtained with 0.3 mg/mL and are omitted in Figure 3.

### 3.2. Brine/CO_2_ Systems

#### 3.2.1. Graphene Oxide (GO)

GO-stabilized CO_2_ foams were studied using the pVT cell. 30 mL of CO_2_ were first charged into the cell. The pressure and temperature were stabilized. Then, 30 mL of GO suspension in SSW (1 mg/mL) were injected into the cell, giving a total volume of 60 mL at the desired conditions. The system was dispersed by stirring for one minute, then the brine and CO_2_ heights were measured. Then, 24 h after charging the cell, the same system was re-stirred using the same procedure and the interphase heights were measured again. This process was repeated also 48 h after the cell charge. Hereinafter, the time specified will always refer to the cell charging time.

**Figure 4:**
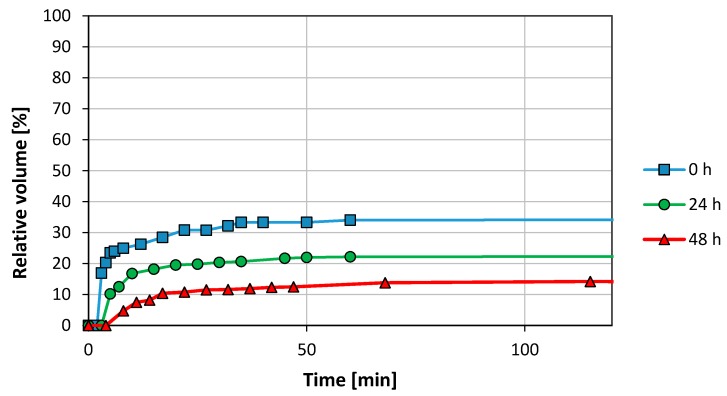
Effect of ageing on foam stability for a concentration of 1 mg/mL GO in synthetic sea water (SSW) (78 bar, 21 °C).

Figure 4 describes the aqueous phase—foam interface at 0 h, at 24 h and at 48 h, as a function of time. The pressure and the temperatures were 78 bar and 21 °C, respectively. The CO_2_-foam interface is not shown since there was not CO_2_ phase segregation. The foam initially observed in the cell was totally opaque and occupied the 100 vol. % of the cell. After 3 min, the appearance of a clear aqueous phase in the bottom of the cell was observed. This phase increased up to 34 vol. % after 40 min, then the aqueous phase volume became constant. In the experiment performed 24 h later, the water profile flatted out approximately after 40 min and was reduced to 22 vol. %. After 48 h, the amount of free water separated was even lower, only 14 vol. %.

After 72 h, the sample was again re-stirred and a significant stability reduction was observed. It was not possible to disperse the particles as effectively as observed during the previous days. Just a few min after stirring stopped, aqueous and CO_2_ phases were separated with a considerable foam phase reduction. Thus, the system stability showed a time-depending effect. Phase volumes were not recorded, however.

**Figure 5:**
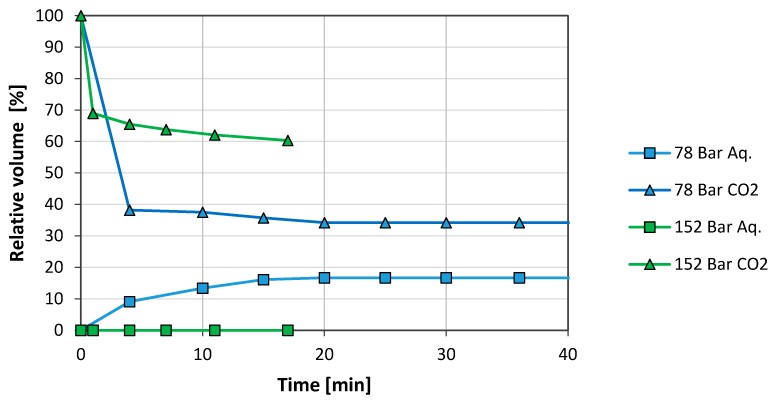
Effect of pressure on foam stability (1 mg/mL GO in SSW, 50 °C). The system had been two days inside the pVT cell prior to the tests.

The cell was next charged with new fluids at the same conditions as before. After two days of ageing (when the previous experiment had shown maximum stability), the temperature was increased from room temperature to 50 °C at constant pressure, 78 bar. Due to the heating, the CO_2_ phase expanded exceeding the 50 vol. % as can be seen in Figure 5. Shortly after, the pressure was increased to 152 bar at constant temperature.

The increased temperature was detrimental to foam stability as observed from comparing Figure 4 at 48 h and Figure 5 (78 bar). After 48 h at room temperature, the system remained stable with 14 vol. % of water separated and the foam phase occupied 86 vol. %. At 50 °C only 16 vol. % of the foam phase remained after 20 min.

The pressure increase improved the foam stability. Water did not segregate and the foam phase stabilised at 60 vol. %.

The time-dependent foam stability was studied further using a lower concentration of particles. The cell was filled with 50 vol. % of each phase at a particle concentration of 0.5 mg/mL at 153 bar. The foam stability was measured after stirring once the sample was injected, after 5 days, after 11 days and 11 days at 226 bar (the system was re-stirred each time).

**Figure 6:**
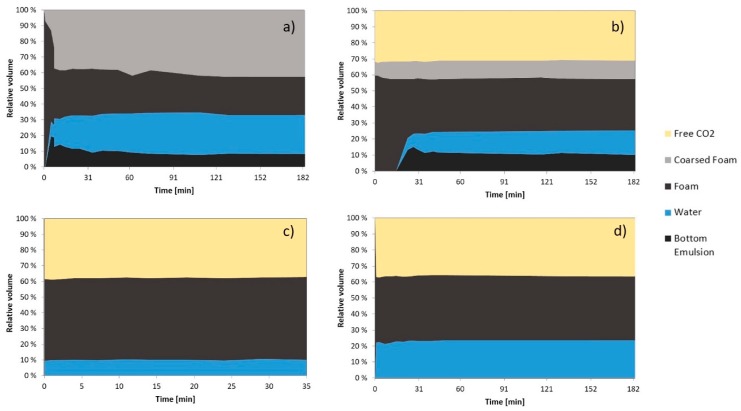
Relative volume of each phase vs. time at 21 °C. The GO concentration was 0.5 mg/mL in SSW. (**a**) Recently made at 153 bar; (**b**) 5 days at 153 bar; (**c**) 11 days at 153 bar; (**d**) 11 days at 226 bar.

**Figure 7:**
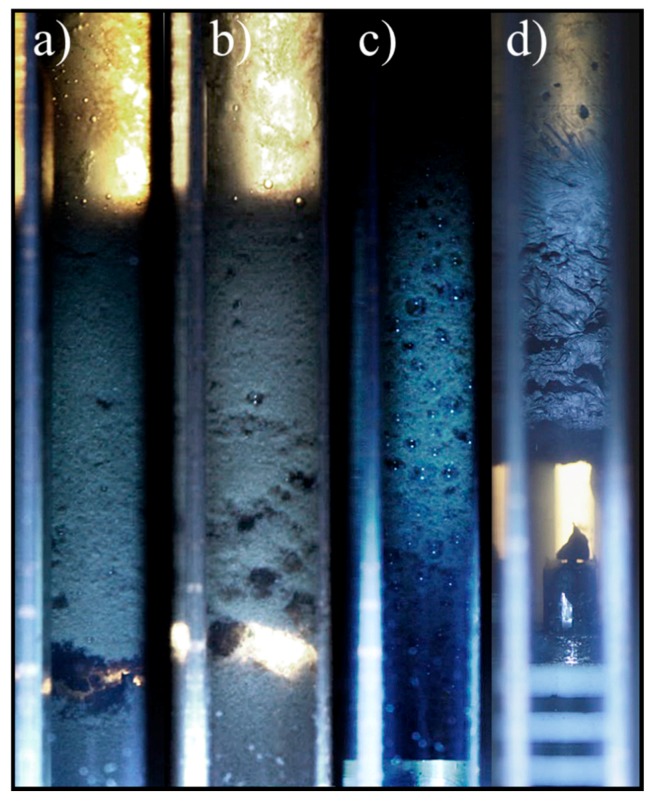
Foam pictures at a different time. (**a**) recently made, (**b**) after 1 h, (**c**) after 3 h and (**d**) after 11 days. The pressure was 226 bar and the concentration was 1 mg/mL in the synthetic brine except for (**d**), where the concentration was 0.5 mg/mL.

The results are shown in Figure 6. When the new system was charged into the cell the formation of free water appeared after 5 min under a large foam phase which immediately started to coalesce. After one hour, the system consisted of 40 vol. % of coarse foam and 40 vol. % of stable foam (Figure 6a). The phase observed under the free water phase was foam that adhered to the piston corners and the magnetic stirrer (Figure 6a,b and Figure 7a,b). After 5 min, the system remained almost unchanged.

After 5 days, the CO_2_ phase appeared immediately after the stirring was stopped (ca. 31 vol. %). The total foam volume decreased to 54 vol. %after approximately 30 min. 10 vol. % was coarse foam and 15 vol. % was free aqueous phase (Figure 6b). After 30 min, only small changes in phase volumes were observed.

After 11 days, the appearance of the foam changed. It adopted a self-folding structure appearance which acted as a plug when re-stirring attempts were made. The system was composed of 37 vol. % CO_2_, 10 vol. % aqueous phase and 53 vol. % of foam (Figure 6c). Then the system was pressurized to 226 bar and a new attempt to stir was made but the foam phase continued acting as a plug.

#### 3.2.2. Foam Morphology

The pVT cell was filled with 1 mg/mL GO in SSW and CO_2_ (50 vol. % of each phase) at 226 bar and room temperature and stirred. The system was re-stirred after 1 h, after 3 h and after 11 days. Pictures of the cell were taken approximately 5 min after each stirring and are shown in Figure 7.

There was an evident change in the foam morphology between the freshly made system and after 3 h. After 3 h the foam phase occupied the whole volume and large trapped CO_2_ bubbles were observed (Figure 7c). Then the system was left in static conditions for 11 days.

Figure 7d shows the system after 11 days for the previous experiments carried out with a concentration of 0.5 mg/mL. However, the systems with 0.5 and 1 mg/mL after 11 days had the same appearance. The 11 days aged system showed a noticeable appearance difference with the systems during the first day (Figure 7a–c). The foam phase exhibited a different morphology, suggesting the formation of a film-like structure.

Once the system was re-stirred, the foam phase acted as a rigid plug not allowing mixing of the phases. Thus, the foam phase had solidified supporting the assumption that self-folding structures were formed.

The increase of stability and its consecutive stability reduction might be the effect of two competitive effects: gelation and precipitation. The presence of divalent ions in the brine could promote system gelation, increasing aqueous phase viscosity and thus, improve stability. Bai et al. [24] already reported the ability of divalent ions (Ca^2+^ and Mg^2+^) to promote hydrogel formation with GO in brine. This hydrogel formation was already observed when the GO was dispersing into SSW. The solution became heterogeneous and the apparition of gel-like aggregates was noticed.

On the other hand, stacking of GO sheets would decrease their interfacial activity and their ability to stabilize dispersed systems. In Figure 7d, particle associations in a film-like structure in the foam phase can be intuited. The stacking of parallel layers is energetically more stable than gel structures due to its larger contact area between GO sheets. This can also be strengthened by the presence of the divalent ions like Ca^2+^ and Mg^2+^ [25]. However, this last stacking mechanism seemed to happen at a lower rate than gelation did.

#### 3.2.3. Partially Reduced Graphene Oxide (rGO)

**Figure 8:**
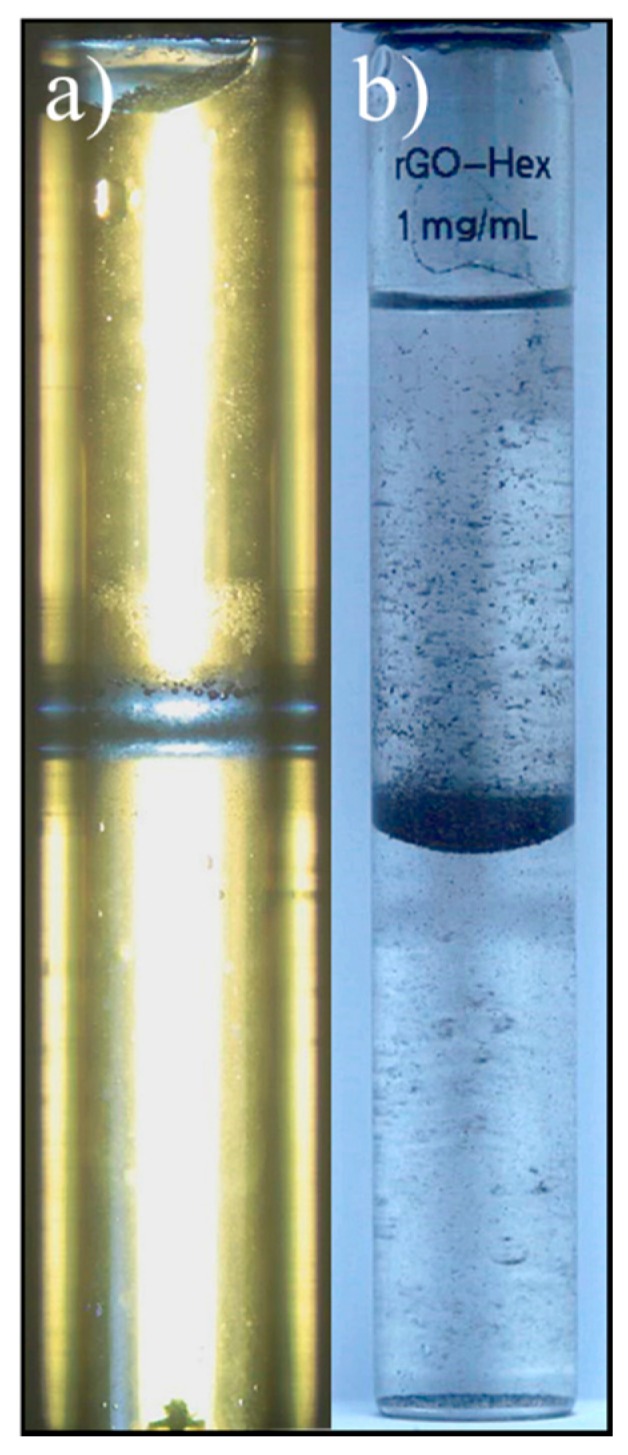
Picture of (**a**) CO_2_/SSW (78 bar and room temperature) and (**b**) n-hexane/SSW.

A CO_2_/SSW system using a concentration of rGO of 1 mg/mL in SSW was studied at 78 bar and 21 °C. However, it was not possible to disperse the CO_2_ into the aqueous phase. Moreover, all particles flocculated and gathered at the interfaces, as observed in Figure 8a.

An attempt to disperse n-hexane into water using 1 mg/mL rGO failed (Figure 8b). It was neither possible to disperse rGO into n-hexane, toluene nor n-decane.

According to Liu et al. [22] reducing the GO increases the CO_2_-philicity of the particles which leads to a better foam stability. However, the present observations are not necessarily opposed to Liu et al. conclusions. In their research, partially reduced GO was used but the reduction degree was not specified nor was an elemental analysis of the particles provided. In this study, the oxygen content was reduced from 41–50% (GO) to 13–22% (rGO). It is reported that ether oxygens can enhance CO_2_ solubility due to Lewis acid—Lewis base interactions with the carbon in CO_2_ [26,27]. It is possible that these particles were more reduced, decreasing the sites for interaction between particles and CO_2_ (epoxy groups) and thus, increasing both their CO_2_-phobicity and the hydrophobicity.

#### 3.2.4. Nanographene Oxide (nGO)

**Figure 9:**
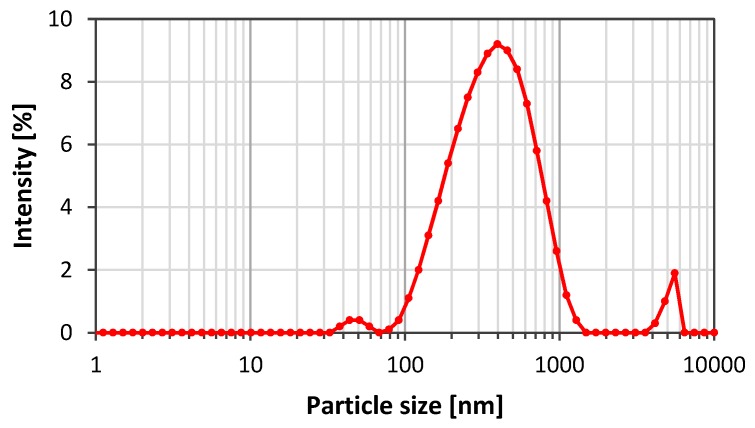
Particle size distribution for nGO particles after ultrasonication and filtration.

The first experiments using GO particles for stabilisation of CO_2_/SSW foam were promising with respect to stabilisation. However, the particles used were large (4 to 30 µm) and the size should be reduced to avoid pore blockage during flow through porous media. Graphenea supplied a reduced particle size nGO suspension in pure water. Figure 9 depicts the nGO particle size distribution. The sample showed a peak around 333 nm and a polydispersity ranging from 70 nm to 1.5 µm. The presence of large particles indicates that particle aggregation occurred shortly after the filtration.

This particle size distribution can be adequate for core flooding experiments. However, it has been reported that GO size reduction increases hydrophilicity and diminishes its ability to adsorb at interfaces [21]. Thus, it was important to test the CO_2_ in brine foams stability for the new batch with reduced size.

The pVT cell was charged with 1 mg/mL nGO dispersion in SSW and CO_2_. The experiments were carried out at 21 °C and at different pressures (100, 150 and 200 bar). The stirring procedure was performed as previously described. For these tests, the volumetric ratio between CO_2_ and SSW was varied from 1:1 to 3:1, which can be within the range for foam stabilised EOR [28,29,30,31,32]. nGO was not able to stabilize CO_2_ foams at any of the studied pressures.

The nGO was found to be too hydrophilic for forming and stabilizing foams, remaining in the aqueous phase and not adsorbing at the CO_2_/SSW interface. A size-reduced GO particle would have a higher density of –COOH and epoxy groups, increasing hydrophilicity and decreasing interfacial activity [21,22]. However, it may be possible to reduce the particles to an optimal degree. At this hypothetic point, the density of –COOH and epoxy groups for a given particle size may reduce hydrophilicity keeping the particles CO_2_-philic enough for interfacial adsorption.

In addition, a filtration test using the initial 1 mg/mL suspension in SSW (heterogeneous suspension as described before) was done using a 1.2 µm cellulose nitrate filter. The filter was blocked immediately. This showed that the particles aggregated in the presence of divalent ions most likely forming hydrogels. Thus, hydrogel formation can stabilise foam, as observed with larger particles but makes the system useless for injection into porous media.

## 4. Conclusions

Large GO sheets can be used effectively to disperse CO_2_ in brine but the particles tested were too large (4 to 30 µm) for flow through porous media. Smaller particles were considered. However, reducing particle size had a determinant effect on foamability. nGO with a particle size below 1 µm was not able to form foams. This can possibly be adjusted by partially reducing nGO to make the particles less hydrophilic but the reduction degree must be carefully considered.

CO_2_ in SSW foams stabilized by GO showed a time-dependent stability. This was likely the result of the competitive effect of two mechanisms, hydrogel formation and GO layer staking. Both mechanisms appeared to be triggered by the presence of divalent ions. Hydrogel formation was the faster mechanism and played a beneficial role for foam stability which initially increased reaching a maximum after two days. Contrarily, GO layer stacking was a slower process and cancelled out the stabilizing hydrogel formation mechanism giving a rigid dispersion of CO_2_ and brine.

Reduced graphene oxide (rGO) with 13–22% oxygen content was not able to form foams. This contradicts the results obtained by Liu et al. [22] and may be due to a high degree of reduction of the particles used. Thus, the degree of reduction must be taken carefully into account as low contents of oxygen would increase both hydrophobicity and CO_2_-phobicity.

It can be concluded that, for the particles tested, rGO appeared to be most hydrophobic whereas nGO appeared to be the most hydrophilic particles. This is in agreement with the findings of Luo et al. [21]. The GO appeared to have an intermediate hydrophilicity and the highest interfacial activity that enabled stabilizing CO_2_ foam.

Hydrogel formation in presence of divalent ions can make graphene oxide particles not suitable for EOR purposes. A dispersion of nGO in SSW could not flow through 1.2 µm cellulose nitrate filters, most likely due to hydrogel formation. Passing the filtration test was set as a requirement for carrying on with core flooding experiments. Even though GO can stabilize CO_2_/SSW foams, the results indicate that they are not suitable for CO_2_ EOR.

## Figures and Tables

**Table 1:** Elemental composition for graphene oxide (GO) and reduced graphene oxide (rGO) (from Graphenea S.A.).

	GO (%)	rGO (%)
Carbon	49–56	77–87
Hydrogen	0–1	0–1
Nitrogen	0–1	0–1
Sulphur	2–4	0
Oxygen	41–50	13–22

**Table 2:** Composition of synthetic sea water.

Salt	Concentration (g/L)
NaCl	23.612
CaCl_2_·2H_2_O	1.911
MgCl_2_·6H_2_O	9.149
KCl	0.746
Na_2_SO_4_	3.407
